# The General Practitioner's Consultation Approaches to Medically Unexplained Symptoms: A Qualitative Study

**DOI:** 10.5402/2013/541604

**Published:** 2012-09-16

**Authors:** Henriette Schou Hansen, Marianne Rosendal, Per Fink, Mette Bech Risør

**Affiliations:** ^1^Research Unit for General Practice, Aarhus University, Vennelyst Boulevard 6, 8000 Aarhus C, Denmark; ^2^Research Clinic for Functional Disorders, Aarhus University Hospital, Nørrebrogade 44, 8000 Aarhus C, Denmark; ^3^Research Unit for General Practice, Department of Community Medicine, University of Tromsø, 9037 Tromsø, Norway

## Abstract

*Background*. The prevalence of medically unexplained symptoms (MUSs) in primary care is about 10–15%. The definition of MUS is descriptive and there are no specific diagnostic criteria for MUS in primary care. Furthermore, a general practitioner's (GP's) categorisation of patients with MUS shows large variation. The aim of the present study is to investigate how GPs employ the definition of MUS and how they manage patients with MUS in daily practice. *Methods*. With a grounded theory approach five focus group interviews with GPs were performed. The interviews addressed how GPs managed MUS and their reflections on the course and prognosis for MUS patients. *Results*. Consultations about MUS develop around the individual patient and usually include several appointments. We identified three different types of consultations: (1) “searching for a disease,” (2) “going by the routine,” and (3) “following various paths.” These types of consultations spanned from a biomedical approach to an approach where multiple explanations were offered to explain the patient's problem. The choice of consultation types was influenced by the GP, the patient and contextual factors which, in turn, affected the diagnostic process. *Conclusions*. A diagnosis of MUS is contextually embedded and the diagnostic process is shaped by the consultation.

## 1. Background 

Many patients in primary care experience physical symptoms that have no demonstrable pathology and cannot be explained by any conventionally defined disease. We name such symptoms medically unexplained symptoms (MUSs) or functional somatic symptoms. Primary care sees a high prevalence of MUS (10–15% of all consultations) [1–3] and provides care for the majority of patients with MUS (about 96%) [4]. At the moment, there are no diagnostic criteria for MUS in primary care [5, 6], and the general practitioner's (GP's) categorisation of patients with MUS shows substantial variation [2].

The medical education prescribes that any diagnosis be rooted in objective signs and organic pathology [7]. Doctors are trained to construct their assessments as a diagnostic process, that is, to collect information and objective signs in their search for a possible diagnosis based on specific diagnostic criteria. However, in their daily practice in primary care, assessments are made within a complex framework where organisational and sociocultural factors, as well as the dynamics of the consultation itself, influence the GP's strategy [8, 9]. Clinical reasoning does not always lead to a clear-cut diagnosis, and the GP as well as the patient may often settle for a less explicit conclusion. This becomes evident during the consultation process concerning MUS, because in the search for a proper management strategy, the lack of objective signs opens up for a negotiation that may be influenced by several factors. Salmon et al. showed that patients with somatising symptoms experienced that they were offered different types of explanations for their symptoms: from rejection to tangible explanations that empowered them [10]. However, empirical research on how patients with MUS are actually managed by GPs in their daily practice remains sparse. 

A sanctioned framework for diagnosing MUS undoubtedly would assist GPs not only in communicating with patients during the diagnostic work-up but also in raising care for these patients to a professional and appropriate level. Furthermore, a sanctioned diagnosis would aid research into this complex phenomenon. The present classification of MUS is ambiguous and controversial [11–13], and the psychiatric classification systems for somatoform disorders have limited applicability in primary care [6]. In order to improve the diagnostic framework of MUS, we need to closely study current clinical practice and make an effort to understand how a diagnosis of MUS would be applied in a primary care patient population.

The aim of this study is to investigate how GPs employ the definition of MUS and how they manage patients with MUS in their daily clinical practice. 

## 2. Methods

We used grounded theory and focus group interviews with GPs. No ethical approval was needed for the study according to Danish Ethical Committees. Participants were GPs only, and the aim of the study was on attitudes and social processes, not biomedical research on humans.

### 2.1. Participants

Using a purposeful sampling approach [14], we invited all GPs from five of 13 counties to participate in the study. The invitation letters were addressed to the GPs' practices. Of those who responded (30–55% of the practices), only few agreed to participate. A total of 28 GPs accepted to join the study. We conducted five focus group interviews from May to August 2006. Each group included three to eight GPs. We invited the GPs from two counties at the same time and conducted these interviews before inviting GPs from other counties. We interviewed each group once. The GPs gave informed consent to participate, they were not paid to take part in the interviews, and they were given no information for preparation before the interviews. 

### 2.2. Focus Group Discussions

All GPs were invited to participate in a two-hour focus group discussion. The main purpose of the interviews/discussions was to address how GPs experienced patients with MUS, how they managed and treated MUS and to obtain their reflections on the course and prognosis for MUS patients. 

For the interviews, we made a questioning round [15, 16], which covered the basic themes: (1) the GPs' understanding of the term MUS, (2) how the GPs were using the term (or similar terms), (3) the ICD-10 criteria for somatoform disorders, and (4) how the GPs managed patients with MUSs and an MUS course typically evolved. Apart from raising the basic themes, the GPs were told a case story about a young girl with multiple visits to her GP. The case was introduced during the focus group interview, not to focus the discussion on one case, but in order to prompt discussion about the definition of patients with MUS in general, and how the GPs themselves could affect the course of these patients in particular. Several other cases were introduced by the GPs via this case. The focus groups were facilitated by the first author (HSH, who is an MD) and observed by one of the coauthors (MR, also an MD or MBR, an anthropologist). 

### 2.3. Analysis

The analysis and the methodology are based on grounded theory [17] and seek to capture the GPs' attitudes and perceptions of the interaction between them and their patients, to understand the conditions and consequences of their management of patients with MUS in their everyday clinic and to gain insight into their reflections and reasoning. 

Each focus group interview was recorded and transcribed verbatim [18]. The two first interviews were studied carefully with an open-minded approach. A detailed line-by-line analysis was applied to generate initial coding and to look for emerging categories. The remaining interviews were then studied using the initial coding. A second study of all the interviews aimed at defining theoretical concepts and categories in order to explain larger data segments. Theoretical coding helped to specify possible relationships between these categories, and the emerging categories were linked together in themes. The different themes were continuously compared throughout the entire analysis to detect similarities and differences and to guide the subsequent analysis. The new categories were used when returning to data codes. The analysis was theory-driven in the sense that derived categories triggered further data sampling (incidents, deviations, i.e.). Long memos were prepared during the reading of the interviews. The memos described analytical reflections, including possible hypotheses and theoretical dimensions, whenever new themes or ideas arose. During this process, the investigator strived to reach an interpretative understanding of patterns, connections, and themes rather than to develop a general theory [17]. The first author performed all coding, categorisation, comparison, and memowriting and outlined the final analysis. Emerging categories and other findings were discussed with all coauthors. During the whole process, the last author (MBR) continuously read the interviews and discussed the coding, themes, and memos produced with HSH. Consensus between the authors on the result of the analysis arose when the analytical outline was deemed consistent with the data according to “fit,” “work,” and “relevance.”

The presented typologies emerged as a result of the use of grounded theory. Such analysis encompasses all data and is not finalised until all theoretical categories have been fully data saturated, that is, when all categories in the entire material have been examined and described in terms of properties and dimensions. The analysis and the final results therefore include differences as well as similarities and disagreements, found during continuous comparison. The results are hence an analytical product representing “types” of management rather than a descriptive presentation of specific data.

## 3. Results 

The analysis focused on how the GPs understand and employ the definition of MUS and how they describe and explain their management of patients with MUS in primary care. 

None of the participating GPs questioned the concept of MUS that was presented to them during the FGD according to our definition mentioned previously. They all recognised patients with MUS as a significant patient group in primary care. Consultations in primary care with an MUS patient develop differently from patient to patient, and they may include several appointments concerning the same health problem. A consultation typically begins with a search for a disease—the biomedical approach—and adopts a broader perspective that includes possible physical, psychological, and/or social perspective, after which it may return and end at its biomedical starting point, or the course may repeat itself. We identified three different types of consultations: (1) “searching for a disease,” (2) “going by the routine,” and (3) “following various paths.” These types of consultations have different foci and deploy different strategies for managing the patient with MUS. The choice of consultation type is determined by the GP, the patient, and contextual factors. 

### 3.1. Searching for a Disease

Consultations where the GP is “searching for a disease” take place within the traditional biomedical approach and the GP uses medical investigations in his or her search for treatable conditions. During these consultations, the GP rarely opens up for psychological or social explanations and usually ignores the patient's psychological or social clues. The biomedical approach builds on the notion that every symptom has a cause that can be found and objectified. MUSs lack positive, objective signs, and in the biomedical approach this fact becomes a constant challenge that calls for action.

*(Discussing the presented case story of a young girl who complains about dizziness and has fainted a couple of times).*

*A (a GP): From this story we might as well learn that in three months' time the patient will suffer from something serious.*

*B (another GP): Yes, it could be epilepsy. It could be it. We do not really think it is, but…*

*A: Right, but you cannot really call it so this without a neurological assessment, but epilepsy, and then it had to be a rare form. I'm not sure they will catch it. Something else that could cause discomfort… *

When the consultation follows the biomedical approach, the GP only addresses the physical aspect of the symptoms. The approach emphasises thorough assessment in order to localise the pathology of a physical symptom.

*Now, for instance, palpitations. It is not enough just to—she may have palpitations. She may suffer from something that means that—you cannot just say palpitations and then no more…She could actually experience some sort of arrhythmia, which she interprets as palpitations, so you should refer her to further examinations or do something else.*

Data show that the GPs use physical examinations, para-clinical tests, and referrals to specialists as their management strategies. They use these strategies in their search for a known disease or in order to exclude a possible differential diagnosis. The GPs express their rationale as a question of needing to know that the patient was properly assessed to begin with in case he/she gets seriously ill later on. 

### 3.2. Going by the Routine

When the consultation is “going by the routine,” the GP also adheres to the basic rules of consultations by examining and diagnosing the patient according to the biomedical approach, but adopting a pragmatic approach, the GP refrains from being dogmatic. The GP only addresses the physical side of the problem, not because the GP is afraid of overlooking a physical disease, but in order to follow the rules of the consultation and make an easy consultation.

*I talk a little around it. Now, I have checked very carefully, I go through the fluid balance, the kidneys and the haemoglobin percentage; and what the blood samples have shown. Very carefully and they are a little increased, so we will check them again in three months, just to make it fade a little. *

The data show that the GP is not unaware of other nonphysical explanations during these consultations. He/she may even think that the problem is psychological or social, but only acts on the physical side of the symptom and does not respond to other cues from the patient. The interventions are not prompted by biomedical rationality alone but are embedded in consultation routines.

*…has pain in his elbow or stomach trouble, which we cannot explain, we cannot find any somatic explanation. Experience tells us that we will see the patient again in a week, and nothing is wrong, they cannot relate to the symptoms. And are happy that we can tell them we have seen this before. We cannot find anything wrong with you, meaning, it will pass by itself.*

The routines are rooted in the situational constraints that often characterize work in general practice, constraints that demand a pragmatic approach—for example, the need to prioritise time, knowing there are more patients in the waiting room. The “going by the routine approach” also resorts to “watchful waiting,” that is, a strategy that allows time to pass before medical intervention or therapy is used and which is adopted because the GP believes that the symptoms are not alarming and may disappear without intervention or therapy. The GPs also express that they wish to avoid “illness” or medicalization—that is, they try to avoid imposing a sick role upon the patient by protecting the patient from tests, and referrals to secondary care, but also by avoiding terms and expressions that can make the patient feel ill. They do so deliberately in the conviction that a normalisation of symptoms is better for the patient than insisting on a confirmation of a disease. During this type of consultation, the GPs do not use diagnoses; they merely note the symptoms. 

### 3.3. Following Various Paths

In those cases where the GPs open up for various explanations right from the start of the consultation, they will simultaneously consider other, that is, physical, psychological, and social, explanations for the symptom and will often do so in an on-going dialogue with the patient. The most obvious physical reasons are checked, but from the beginning the GP has alternative explanations in mind, and he/she will talk to the patient about various topics like work, family, mental wellbeing, and so forth.

*And then, usually, I “ride two horses” when explaining the model. I say that it is reasonable to examine you for this and that, even though I still think that the main cause is something psychological, for example, anxiety or something similar. Then we can examine you for this and that, and when you have been examined then we make a proper assessment. You see, that is the way to do it. *

When choosing “the other horse,” the GP deploys an approach that seeks a psychosocial explanation of a more complex character than the biomedical approach. Physical assessments are made, but mainly to reassure the patient. The interviews made it clear that this approach presupposes the patient's cooperation. It is assumed that the patient will accept the complexity of the problem and is willing to open up for other explanations than the physical one. The patient and the GP must negotiate to reach a shared understanding of the problem, and this mutual understanding must be upheld during the management of the problem. In this perspective, effective management requires that the GP establishes an alliance with his or her patient. The GPs use tests and referrals as a strategy when making an alliance with the patient, thereby using the same strategy as in “searching for a disease.” At any time, the diagnosis as well as the explanations of the problem is being continuously negotiated and may be questioned by both the patient and the GP, which opens up for other approaches.

*To me it is the ideal situation when someone turns to me with such matters. Then the patient and I will find out together that it is something else that is happening, besides the physical aspect, and this takes two. The “somatic” defects we can handle on our own, but this we must solve together with the patient, since this is how you solve this problem and find the right direction for solving it.*

In this approach, educating the patient is also considered an important management strategy. Educating here means teaching the patient to be aware of the signals from his or her body and helping the patient to manage his or her symptoms. Teaching the patient basic knowledge about the body is also found to be helpful. Basically, in this consultation approach, the GPs recognize that the symptoms presented to them are sometimes bordering on normal reactions to distress and that not everything has a biomedical explanation, and thereby they open up for more complex explanations. 

### 3.4. The Pattern of Consultations

According to the interviewed GPs, the different consultation typologies described previously represent different approaches that may each be deployed at variable time points in the series of consultations of a patient with MUS. The three types of approaches form a consultation circle and are not to be seen as ultimate or singular approaches, but as three different options within this circle, that is, options that may be chosen, returned to, or shifted between. Over a period of time, the patient with MUS will have several contacts with the GP, and resort will be made to the various types of consultations to solve the problem. Hence, the MUS diagnosis is rarely made at one consultation. All GPs use all three consultation types, but which type they choose depends on the patient, the GP, and situational factors such as time. Our interviews show that during every consultation and before the GP starts to search for other explanations, the GP rules out the most obvious physical diseases as a precaution, as a routine, or out of professional conviction. All doctors will reason according to the diagnostic process: history-taking, objective assessment, diagnosis, and treatment. However, the doctors need to base a diagnosis on positive signs, but what is accepted as a positive sign differs. These differences reflect diversity in the understanding of the problem, and the management strategies chosen therefore often diverge.

*Until they have had it clarified and confirmed [if a symptom is physically explainable], there is a theoretical possibility of the patient being right. We may then communicate better, I think, and it may be easier to reach a mutual solution. *

“Searching for a disease” is used to reassure the patient or the GP him/herself that nothing has been missed, and the GP will return to this strategy when checking symptoms, just to be safe. GPs who use this strategy do not think that the problem is MUS: they are more or less convinced that the presented symptoms have a pathophysiological aetiology and they therefore search for an objective sign of the symptom in the belief that its cause can be found. If the patient keeps coming back with the same problem and cannot be reassured, or if the patient or the problem is new to the GP, this could also encourage the GP to use the strategy “searching for a disease.” Most GPs will use “searching for a disease” as their first diagnostic approach. Hence, it was considered a problem that patients with MUS would not always see the same GP in partnership practices because this would make it more likely that they would have more tests than necessary. 

At consultations that are “going by the routine,” the symptoms are not found to be alarming, and the GPs will use their experience and expect the symptoms to pass within a short time.

*I am now better at telling people that nature will take care of this, and if it has not done this within two weeks, let us talk again. In that respect, you can change over time. *

The GPs see symptoms as a part of life; symptoms come and go and many symptoms will remain unexplained. GPs consider this approach a time saving one and think that following other leads than the physical one is time-consuming and therefore not always a realistic possibility. The GPs also acknowledge that they sometimes just do not perceive what the patient tells them, do not take the time, or are not interested in searching for more complex explanations. The GPs expressed that they need to have some kind of sympathy for the patient to embark on a strategy of “following various paths.” Sometimes, the patient only wants to discuss the physical side of the problem, and in these situations “going by the routine” can be used as a nonconfronting approach deployed not only to save time, but in some cases also to help build a future alliance with the patient. 

“Following various paths” is often used when the GP knows the patient, either through several consultations within a short time or because the GP has known the patient for many years. A therapeutic alliance is often seen as a precondition for this approach. The GPs do not see the patients as being ill, merely strained. The strain can be either permanent or transient and can be physical, psychological, or social. The patient may open the consultation by identifying a psychosocial explanation, and the GP may accept this as a reasonable explanation. Sometimes, knowledge of obvious psychological or social distress in the patient's life makes it clear that the explanation to the problem is complex. 

Setting the boundaries that define when a patient has been properly assessed and defining when the time is ripe for seeking other, nonphysical explanations for symptoms seem to be a personal issue for the GP. “Following various paths” is an approach that is much more individual than “going by the routine” or “searching for a disease,” both because the GP sets boundaries for physical assessment and because the GP must find his/her own management and treatment strategy, whereas “going by the routine” and “searching for a disease” lie closer to the strictly biomedical approach and the medical education. Some GPs consider “following various paths” to be the ideal consultation form; every consultation, they argue, should be like this. They find the strategy easy to employ and are comfortable in the interaction with the patients. Other GPs prefer to use a more traditional, biomedical approach, and they will only consider other explanations when all other options have been tested. Depending on the GP's understanding of MUS and distress in general, the preferred consultation type will reflect what the GP accepts as a diagnosis. 

## 4. Discussion 

### 4.1. Summary of the Main Findings

The diagnosis of medically unexplained symptoms is not one that can be made out of context, and the diagnostic process of MUS is influenced by the consultation approach. The GP's choice of approach for patients with MUS varied from consultation to consultation, and the patient had usually gone through a series of consultations that focus on the physical aspects of the problem before the GP addressed the possibility of the symptoms being medically unexplained. The approaches could vary from a biomedical approach to a more biopsychosocial approach, but the GPs would exclude the most obvious physical diseases before looking for other explanations. 

### 4.2. Strengths and Limitations of This Study

The purposeful sample of participants provided in-depth information about issues and themes concerning the GPs' understanding of MUS and how they managed patients with MUS. Practical constraints determined the sample size. We reached a point in the data analysis where new categories revealed no new theoretical insights. 

None of the participating GPs questioned the concept of MUS. They all recognized patients with MUS as constituting a significant patient group in primary care. It should be kept in mind, though, that our GPs may have been biased when they accepted participation, and their decision to participate in the study may testify to a relatively positive attitude towards patients with MUS. We have no data on how GPs who did not accept the invitation view this concept and manage this patient group. Salmon et al. have shown that GPs who declined training in reattribution techniques were devaluating their psychological skills and had a more negative attitude towards patients with MUS than participating GPs [19]. 

We wanted to obtain information about the GPs' use of the concept of MUS in their verbal expressions and self-reported data by means of FGD [20], but we are aware of the risk that participants expressed their ideal attitudes. However, FGDs are helpful when exploring complex issues, and listening to others' experiences may help the participants to articulate and organise their own views. The interaction between participants may open up for arguments the participant would not have thought of, if not for the discussion in the focus group.

The facilitator often challenged opinions and statements that were brought up during the FGDs by inviting others to respond, by asking “why” or by giving provocative counter examples of situations and patients. This was done to invite various views on the topic. However, even though the GPs were invited to discuss patients with MUS in general, they tended to confine their talk to complex cases where they see and master the complexity involved. This meant a reduced focus on more problematic cases, but we do not believe that it excluded variation within MUS cases, as many different cases were raised in the discussion by the GPs themselves. During the FGDs we found that referring to the tradition of a holistic patient view, the GPs often tried to be good doctors for patients with MUS. Therefore, noncomplex cases, where the GPs only attended to the physical side of the problem, may not have received much focus during the interviews as would be warranted by their occurrence in everyday practice. Other studies have shown a discrepancy between clinical practice and reported attitudes and beliefs and that a positive attitude towards a condition or a belief in one's skills may not necessarily reflect the corresponding or expected behaviour [21]. With the above in mind, it could have been useful to supplement the study with an observational study of GPs and patients with MUS that would have supplemented the present findings with findings from actual clinical practice. An observational study could also provide information on factors influencing the GPs' consultation approaches. 

### 4.3. Results in relation to Other Studies

The consultation approaches described in our study may be seen as general consultation processes, and the first consultation with a patient with MUS will usually be the same as with any other patient since the GP needs to rule out any obvious disease. The interesting issue is why and how the GP switches from one approach to another. A Dutch study showed that three different models for alliances are used to explain MUS to a patient and to promote maintenance of the doctor-patient relationship, with varying consequences for care and treatment [22]. The GPs in the present study also mentioned that they used different practices and approaches. Embedded in this discussion is the issue of how to combine the biomedical foundation with experience-based learning and situational knowledge. Engel's introduction of the biopsychosocial model are illustrates that there is more to medicine than biomedicine [23].

In the interviews, MUS was rarely diagnosed in one consultation, but emerged over time as the result of several consultations. These consultations could each represent different approaches, and the diagnostic process was shaped by the consultation approaches used. This corresponds well with the discussion about decision making in primary care, where it is generally recognized that the diagnostic decision is made within a complex framework [8, 9, 24]. 

Our GPs used different kinds of explanations and definitions of MUS at different times, and this is reflected in the approaches they adopted. This observation tallies with findings in patient interviews where patients with MUS encounter different kinds of explanations when consulting the doctors [10, 24]. 

Depending on the chosen approach, the GPs will be open or closed to psychosocial cues, consider engagement in psychosocial discussions as time consuming, and/or need to feel sympathy with the patient to make the extra effort. This is in accordance with observations made by Ring et al. who found that GPs seldom engage in psychosocial cues, and cues from the patients are rarely picked up [5]. Salmon describes that the GP's willingness to address emotional cues varies depending on the GP's mood and work pressure [24]. 

In the focus groups, the GPs talked about tests and referrals as a decision made together with the patient. Sometimes, the patient would be more firm than at other times, and if the patient did not want to discuss other explanations than the physical one, this could make the GP choose a more biomedical approach than he/she originally wanted. This was not described as being put under pressure like in other studies [9, 25, 26]. Salmon described that the longer the consultation, the larger the risk of an intervention, for example, a test or referral [27]. The GPs in our focus groups did not raise the issue of the duration of the consultation, but they raised the issue that occasionally the patient could not be reassured, or they could not find an explanation for the problem together with the patient. They would then turn to a biomedical approach. Ring et al. showed that physical interventions are suggested more often by GPs than by patients [5, 28]. This is consistent with our data where the GPs often used the strict biomedical approach with tests and referrals as management strategies. 

## 5. Conclusions 

 This study identified three different approaches to patients with MUS. The GP's diagnosis and management of patients with MUS depended on the approach taken: “searching for a disease,” “going by the routine,” or “taking various paths.” The diagnostic process of MUS is influenced by the consultation approach and cannot be analyzed out of context. However, the results of the present study are based on the GPs' statements about their consultations, and future research should further explore GPs' actions and patients' experiences. Observational studies in primary care would contribute to our knowledge about when and why the described consultation approaches are chosen and how the GPs switch from one approach to another. Such knowledge would support and potentially improve GP diagnostics and management of patients with MUS in the future.
